# Does physicians’ right to strike outweigh students’ right to an education? The on-going ethical dilemma in Peru

**DOI:** 10.3402/meo.v17i0.19870

**Published:** 2012-12-19

**Authors:** Rodrigo M. Carrillo-Larco, Álvaro Taype-Rondán, Reneé Pereyra-Elías

**Affiliations:** 1 Sociedad Científica de Estudiantes de Medicina Cayetano Heredia (SOCEMCH), Lima, Perú; 2 Sociedad Científica de Estudiantes de Medicina de la Universidad de San Martín de Porres (SOCIEM-USMP), Lima, Perú; 3 Escuela de Medicina, Universidad Peruana de Ciencias Aplicadas, Lima, Perú

Although often viewed as an action of last resort, going on strike remains a legal and often effective option for physicians seeking labor improvements and better working conditions. Indeed, in some countries, there have been reports of strikes by physicians (1, 2), followed by ensuing discussions of potential ethical implications (3–5). However, little has been said about the consequences of such a mass labor stoppage on undergraduate medical education – and those students who aspire to the profession.

In Peru, physicians from the Peruvian National Social Insurance (EsSalud) went on a 33-day strike (August 7 to September 8), effectively limiting medical services to only emergency and critical care units. Furthermore, per EsSalud's labor guidelines (prepared for purposes of the strike), all academic activity within affiliated teaching hospitals was explicitly forbidden during the strike.

Although some universities tried to reschedule classroom activities, reorganizing clinical education on such short notice left few options. As a result, due to EsSalud having numerous teaching hospitals nationwide (6), many students saw their classes or clinical training abruptly postponed and, in some cases, even canceled. In Peru, this situation was especially problematic for sixth-year (pre-internship) students needing to complete a certain amount of practice hours to be promoted to interns.

Perhaps ironically, many Peruvian medical students may well support the striking physicians’ demands (e.g., better working conditions and salaries, improved care for patients) – and perhaps even their decision to voluntarily suspend medical services. In other countries, medical students who have found themselves in similar situations have considered striking as a valid option in order to achieve the physicians’ demands (7, 8). Whether or not Peruvian medical students fully supported the strike, it certainly affected the educational activities in the EsSalud teaching hospitals:

1. All hospital-based classes (both classroom and clinical) were suspended.
2. Students who went to the hospitals to discuss clinical cases or topics with their teacher (without access to patients) were promptly asked to leave the facilities – forcing them to seek out other university spaces (or by gathering in coffee shops).
3. In some facilities, students missed rotations have yet to be rescheduled – making it unclear if they will be made up.
4. To our knowledge, at least one medical school was forced to cancel courses until next year due to the strike.

The strike, for several reasons, obviously resulted in fewer patients clearly impacting upon educational endeavors. However, it is also possible that some academic activities could have been sustained during the strike.

For instance, some practical classes could have been sustained in those services that remained active, such as emergency consults, emergency operation rooms, or the intensive care unit. Likewise, classroom activities and discussions might have been carried out in non-patient consultation rooms, offices, or other available venues. Such gestures would not necessarily have meant that those professors were unsupportive of the strike. It could have reflected a commitment to their teaching and mentoring responsibilities.

More recently, there was another nationwide strike by doctors working for the Ministry of Health (MINSA), in effect from September 18 to October 20. Labor guidelines also explicitly prohibited certain teaching activities here. Again, the situation placed some medical students in a potentially damaging situation, begging the question: Is it ethical for physician educators to suspend medical training activities during an organized labor strike?
